# Introducing Decision Aids into Routine Prostate Cancer Care in The Netherlands: Implementation and Patient Evaluations from the Multi-regional JIPPA Initiative

**DOI:** 10.1007/s13187-019-01572-9

**Published:** 2019-07-05

**Authors:** Maarten Cuypers, Hoda H. M. Al-Itejawi, Cornelia F. van Uden-Kraan, Peep F. M. Stalmeier, Romy E. D. Lamers, Inge M. van Oort, Diederik M. Somford, Reindert Jeroen A. van Moorselaar, Irma M. Verdonck-de Leeuw, Lonneke V. van de Poll-Franse, Julia J. van Tol-Geerdink, Marieke de Vries

**Affiliations:** 1 Department of Social Psychology, Tilburg University, Tilburg, The Netherlands; 2 Radboud Institute for Health Sciences, Department of Primary and Community Care, Radboud University Medical Center, Nijmegen, The Netherlands; 3 Department of Urology, VU University Medical Center, Amsterdam, The Netherlands; 4 Department of Clinical Psychology, VU University, Amsterdam, The Netherlands; 5 Radboud Institute for Health Sciences, Department for Health Evidence, Radboud University Medical Center, Nijmegen, The Netherlands; 6 Department of Urology, Elisabeth-Tweesteden Hospital, Tilburg, The Netherlands; 7 Department of Urology, Radboud University Medical Centre, Nijmegen, The Netherlands; 8 Department of Urology, Canisius Wilhelmina Hospital, Nijmegen, The Netherlands; 9 Department of Otolaryngology/Head and Neck Surgery, VU University Medical Center, Amsterdam, The Netherlands; 10 CoRPS—Center of Research on Psychology in Somatic Diseases, Department of Medical and Clinical Psychology, Tilburg University, Tilburg, The Netherlands; 11 Department of Research, Comprehensive Cancer Organisation Netherlands, Eindhoven, The Netherlands; 12 Department of Psychosocial Research and Epidemiology, Netherlands Cancer Institute, Amsterdam, The Netherlands; 13 Department of Radiation Oncology, Radboud University Medical Center, Nijmegen, The Netherlands; 14 Institute for Computing and Information Sciences (iCIS) & Social and Cultural Psychology, Behavioural Science Institute, Radboud University, Nijmegen, The Netherlands

**Keywords:** Prostate cancer, Oncology, Shared decision-making, Decision aids, Implementation

## Abstract

Uptake of decision aids (DAs) in daily routine is low, resulting in limited knowledge about successful DA implementation at a large scale. We assessed implementation rates after multi-regional implementation of three different prostate cancer (PCa) treatment DAs and patient-perceived barriers and facilitators to use a DA. Thirty-three hospitals implemented one out of the three DAs in routine care. Implementation rates for each DA were calculated per hospital. After deciding about PCa treatment, patients (*n* = 1033) completed a survey on pre-formulated barriers and facilitators to use a DA. Overall DA implementation was 40%. For each DA alike, implementation within hospitals varied from incidental (< 10% of eligible patients receiving a DA) to high rates of implementation (> 80%). All three DAs were evaluated positively by patients, although concise and paper DAs yielded higher satisfaction scores compared with an elaborate online DA. Patients were most satisfied when they received the DA within a week after diagnosis. Pre-formulated barriers to DA usage were experienced by less than 10% of the patients, and most patients confirmed the facilitators. Many patients received a DA during treatment counseling, although a wide variation in uptake across hospitals was observed for each DA. Most patients were satisfied with the DA they received. Sustained implementation of DAs in clinical routine requires further encouragement and attention.

## Introduction

Prostate cancer (Pca) is the most common malignancy diagnosed in men in the western world. In the case of localized prostate cancer, patients are typically required to choose between multiple equivalent treatment options. Although survival perspectives with each treatment are similar, treatment procedures and risk for side effects vary, and many patients have poor understanding of these differences between treatments [1]. Therefore, clinical guidelines concerning localized Pca suggest a shared patient-doctor decision to incorporate patient preferences and values into the treatment decision [2–5]. Decision aids (DAs) have been developed to assist patients and care providers with shared decision-making (SDM) [6].

Evidence for the beneficial effects of applying DAs is widely available and shows that patients have better knowledge of the treatment options, and are more aware of their personal preferences and values [7]. As a consequence, DAs help patients to take a more active role in the decision-making process [8]. So far, most DA trials, including those related to Pca treatment, focused on determining the DA effects, with limited attention for implementation aspects [7, 9]. Many DA trials took place within a single institution or location, and even if the absolute number of the DAs distributed was known, their relative reach within the targeted patient population often remained unknown [10, 11]. Moreover, uptake of DAs in daily routine, outside of clinical trials, is low, resulting in limited knowledge about successful DA implementation at a large scale [7, 12–16].

After distribution of the DA to eligible patients, the next step in implementation is actual DA use by patients. Patient-perceived barriers and facilitators related to DA usage have been studied more extensively [12, 17–21]. Common barriers against DA usage from the patients’ perspective are insufficient trust in the DA quality or its benefits, the DA being unpractical in use, inadequate timing (e.g., the DA being offered too late after diagnosis), or inadequate explanation of how to use the DA. Patient-perceived facilitators include that the DA is practical in use, and that the presented information is complete and trusted [12, 17–21].

This study was conducted by the Joint Implementation Prostate cancer Patient-centered care (JPPPA) consortium, consisting of three DA research groups that each developed a DA for Pca patients. With the current implementation study, we aimed to investigate the implementation rate of these three DAs in routine Pca care in The Netherlands, and aimed to identify possible barriers and facilitators from the patients’ perspective.

## Patients and Methods

### The Three Decision Aids

Each of the three DAs involved was developed according to the International Patient Decision Aids Standards (IPDAS) and contained information about the disease, treatment options, and (dis) advantages of all options based on (inter) national guidelines and international literature [22]. Patients, urologists, and radiation oncologists were involved in the development and review process of the DAs [23–25]. In each DA, the same choice options were presented: surgery, brachytherapy, and external beam radiotherapy, as well as the option of active surveillance. The DAs varied in their format and length. DA1 was a concise booklet (14 pages), DA2 was an even more concise (in diagram style with short explanations) booklet or online DA (by patient choice), and DA3 was an elaborate online DA with values clarification exercises (VCEs). The DA format coincided with the intended moment of use. DA1 and DA2 could be incorporated in clinical consultation, or used at home, while DA3 was, by design, supposed to be used outside of consultations. The characteristics of the DAs are presented in Table 1. Detailed descriptions of the separate trials investigating the DA effects have been published earlier [23–26].

**Table 1:** Characteristics of the three DAs

	DA 1	DA 2	DA 3
			
Implementation period	July 2013–July 2014	March 2014–March 2016	August 2014–June 2016
Number of hospitals	8	16	8
Number of DAs distributed	284	273	351
Number of patients evaluating DA	255	183	235
DA format	Print booklet	Print booklet or online (by patient choice)	Online
Intended use	During consultation	Outside consultation	Outside consultation
DA content	General information about (treatment of) Pca is described first, then specific information on the procedures, the likelihood of cure and side effects in the urinary, and bowel and sexual domain for the each treatment is described. Risk information on the probabilities of progression, survival, and side effects (urinary, bowel and erectile) are presented by means of pie charts. No explicit values clarification exercises are included.	Treatment options are described in short terms. Arguments in favor and against each treatment are presented separately. Pros and cons that are presented include the following topics: cure, treatment, and quality of life. No explicit values clarification exercises are presented. An alphabetical glossary of difficult terminology is included, adjusted to low literacy. No values clarification exercises are included.	Elaborate information (text and graphics) about Pca and common terminology is provided. Active surveillance is compared with treatments, and in a next step, surgery is compared with radiation options. Advantages, disadvantages, and risks of each option are discussed. Risks are presented in a graphical display. VCEs are included as statements to trade off treatment attributes. A DA summary can be obtained for use during a follow-up consultation.

### Setting and Participants

Thirty-three hospitals (out of a total of 90 hospitals in The Netherlands) implemented one of the three DAs in treatment counseling. Each DA was implemented in a specific region of The Netherlands (DA1 East, 8 hospitals; DA2 North-West, 16 hospitals; DA3 South, 9 hospitals). Per DA, hospitals were recruited to participate based on convenience (e.g., distance); the allocation of DAs to hospitals was not randomized. The DAs were handed out to patients newly diagnosed with localized Pca. For all 3 DAs, patients were eligible to participate if they had the possibility to choose between at least two treatments covered by the DA. The assessment of whether the DA was applicable (e.g., eligibility for at least two treatments covered by the DA) was done by the patient’s urologist. Actual distribution of the DA was done by either the urologist or a specialized nurse, depending on what best fitted with existing local care pathways. After the treatment decision was made, but before treatment started, patients received a questionnaire to evaluate receipt and usage of the DA. All data were collected between July 2013 and June 2016. Research protocols from each DA group were reviewed by their respective local institutional ethics committees, which each provided a waiver for further ethical assessment.

### Outcome Measures

Our primary outcome measure was the implementation rate. This rate was calculated by the proportion of patients who received a DA compared with the estimated total number of eligible Pca patients per hospital during the period the DA was implemented. Since the total number of eligible patients was not prospectively registered in a structured manner in all participating hospitals, an estimation was based on hospital-specific registry data of the 6 years prior to the current project, retrieved from the Netherlands Cancer Registry.

After a treatment decision was made, a questionnaire was used to collect self-reported data about patient’s demographic variables (age, marital status, having children, and educational level). Evaluation measures consisted of DA distribution procedures (e.g., “Who presented the DA to you?”), DA user-friendliness (e.g., “Did it occur fonts were too small?”), and a 24-item list of barriers and facilitators for DA use (e.g., “I had insufficient trust in the DA”) based on literature [21] (items presented in Table 3). All three DA groups used the same questionnaires to evaluate DA use in order to enable combined data analyses.

### Data Analysis

Descriptive questionnaire data are presented as means (Ms) with standard deviations (SDs) for continuous variables, and frequencies and percentages for categorical variables. Comparisons between DAs for continuous variables were made with analyses of variance (ANOVA) and Bonferroni post-hoc tests and with chi-square tests for categorical variables. Statistical analyses were conducted with SPSS 22.0 (Statistical Package for Social Sciences, Chicago, IL). Tests were two-sided and considered statistically significant if *p* < .05.

## Results

**Fig. 1:**
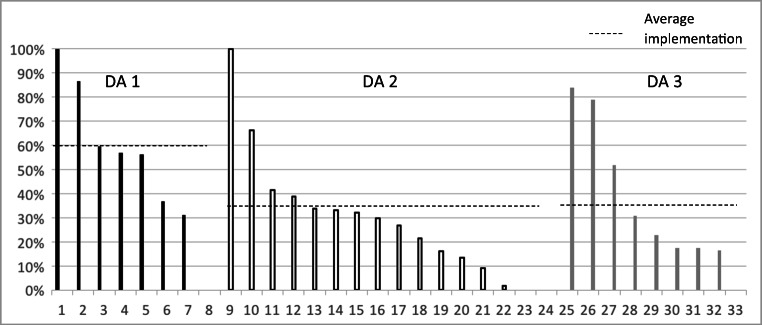
Implementation rates per hospital (*n* = 33)

During the study period, 908 newly diagnosed Pca patients received a DA out of an estimated total of 2285 eligible patients, resulting in an overall implementation rate of 40%. With each DA, high implementation levels (> 80%) were achieved in 1 or 2 hospitals, whereas for the other hospitals, implementation varied considerably (2–80%). The highest average implementation was achieved with the concise paper DA1 (60%); average implementation levels for DA2 and DA3 were comparable (34–35%). Implementation rates across hospitals for each DA are presented in Fig. 1.

Out of the 908 patients who received a DA, 673 patients agreed to complete the post-decision questionnaire evaluating DA use (response 74%). Compared with participants from both other DA groups, participants from DA3 were slightly younger and more often highly educated (Table 2). Mean PSA and Gleason scores were lower for participants from DA3, but the same distribution across categories was found between DA groups (Table 2).

**Table 2:** Sociodemographic and clinical characteristics of questionnaire responders

	DA 1 (*n* = 255)	DA 2 (*n* = 183)	DA 3 (*n* = 235)	*p*
Age at informed consent, mean (SD)	66.0 (5.9)	66.3 (6.2)	64.9 (6.0)	.04
Marital status, *n* (%)				
Married/living together	222 (87%)	149 (81%)	208 (88%)	.09
Single/Other	33 (13%)	34 (19%)	27 (12%)	
Education, *n* (%)				
Low	94 (37%)	63 (34%)	76 (33%)	.01
Medium	62 (25%)	66 (36%)	54 (23%)	
High	96 (38%)	54 (30%)	101 (44%)	
Gleason score, mean (SD)^1^	6.5 (0.7)	6.7 (0.9)	6.4 (0.8)	.001
≤ 6, *n* (%)	158 (63%)	89 (53%)	134 (61%)	.13
≥ 7, *n* (%)	93 (37%)	78 (47%)	86 (39%)	
Missing, *n*	4	16	15	
PSA level, mean (SD)^1^	9.2 (5.3)	9.9 (8.4)	7.9 (3.9)	.002
≤ 10.0, *n* (%)	183 (73%)	115 (69%)	180 (77%)	.20
10.1–20.0, *n* (%)	60 (24%)	42 (25%)	49 (21%)	
≥20.1, *n* (%)	8 (3%)	10 (6%)	5 (2%)	
Missing, *n*	4	16	1	

*p* values report comparisons between trials for the control groups and DA groups, according to *t* tests and analysis of variance (ANOVA) for means and *χ*^2^ tests for frequencies

Numbers may not always add up to the same *n* due to missing data (e.g., item non-response); percentages are rounded

Scores of participants from DA1 and DA2 were obtained from medical records; DA3 presents self-reported scores

Most participants indicated that they received the DA from their urologist (*n* = 478, 71%) and perceived that the urologist is the most suitable person to hand out a DA (*n* = 511, 76%; Table 3). However, of the participants who received the DA from a nurse (*n* = 192, 29%), 60% considered the nurse to be the most suitable person for this (data not shown). Most participants (*n* = 573, 85%) perceived that they received sufficient explanation about the DA, regardless of DA type, care provider handing out the DA (urologist versus nurse), or moment of receipt (Table 3). Almost all participants who used DA1 or DA2 were satisfied with the DA format (99 and 96%), but for the online DA3, a considerable proportion of participants (*n* = 67, 21%) would have preferred a paper format (Table 3). Overall, satisfaction with the online DA3 was lower compared with DA1 and DA2 (Table 3).

**Table 3:** Patient DA evaluations and barriers and facilitators

	DA 1 *N* = 255	DA 2 *N* = 183	DA 3 *N* = 235	*p*
Practical implementation, agreed with statement, *n* (%)				
Received DA from doctor	189 (78%)	138 (76%)	**151 (64%)**	.003
Doctor is most suitable to provide DA	200 (82%)	143 (81%)	**168 (72%)**	.02
Received DA within a week from diagnosis	175 (69%)	**159 (87%)**	154 (66%)	< .001
Satisfied with moment of receipt	232 (92%)	173 (95%)	196 (92%)	
DA was sufficiently explained	226 (89%)	161 (88%)	186 (87%)	
Satisfied with DA format	250 (99%)	176 (96%)	**168 (79%)**	< .001
DA added much to other information	181 (83%)	141 (83%)	**107 (56%)**	< .001
Implementation barriers confirmed, n (%)				
Forgot to use the DA	6 (2%)	4 (2%)	9 (4%)	
DA was too difficult	7 (3%)	3 (2%)	10 (5%)	
DA was steering towards a treatment	21 (9%)	14 (8%)	20 (10%)	
DA was unclear	5 (2%)	9 (5%)	12 (6%)	
DA was unpractical	10 (4%)	9 (5%)	**25 (12%)**	.002
Was not confident in DA	20 (8%)	8 (4%)	24 (12%)	.03
Expected no benefit	15 (6%)	15 (8%)	**29 (14%)**	.01
Expected DA would be burdensome	12 (5%)	4 (2%)	11 (5%)	
Not motivated to use DA	11 (5%)	4 (2%)	13 (6%)	
Expected DA would increase uncertainty	17 (7%)	5 (3%)	13 (6%)	
DA was insufficiently adjusted to specific needs	30 (12%)	**8 (4%)**	28 (14%)	.006
Implementation facilitators confirmed, n (%)				
DA was pleasant to use	223 (91%)	166 (91%)	**166 (80%)**	.001
DA was well organized	234 (95%)	172 (94%)	**175 (85%)**	< .001
DA enabled treatment comparisons	222 (90%)	164 (90%)	**163 (79%)**	.001
DA gave insight in treatment (dis)advantages	226 (92%)	170 (93%)	**168 (81%)**	< .001
Felt DA information was complete	204 (84%)	154 (84%)	**154 (74%)**	.02
DA was important addition to other information	217 (90%)	166 (91%)	**152 (73%)**	< .001
Pleasant to use DA as additional source of information	231 (94%)	160 (87%)	165 (80%)	< .001
Confident in DA quality	231 (94%)	170 (93%)	**170 (82%)**	< .001
Expected DA would reduce uncertainty about decision	167 (69%)	**146 (80%)**	124 (60%)	< .001
Used the DA to determine treatment	176 (72%)	153 (84%)	123 (59%)	< .001
DA made easier to talk with relatives	202 (83%)	160 (87%)	**129 (62%)**	< .001
DA made easier to talk with care providers	196 (81%)	157 (86%)	**123 (59%)**	< .001
Recommend DA to others	219 (100%)	171 (99%)	**172 (90%)**	< .001

Percentages are calculated based on item response, not as a proportion of the group total presented in table header

*p* values represent the outcomes of chi-square tests comparing all three DAs; significant differences caused by a single DA are indicated in boldface

Barriers against DA usage were reported by less than 10% of the participants, regardless of which DA they received (Table 3). Differences found between DAs were related to format (unpractical, insufficiently adjusted to personal preferences) or subjective evaluations (no confidence, expected no benefit). Overall, most barriers were reported for the elaborate online DA3.

Facilitators for DA use were reported by a large majority of participants (Table 2). For all DAs, more than 80% of participants found the DA pleasant to use and well organized and were confident in the DA quality. Overall, facilitators were reported mostly by respondents who used the most concise DA (DA2) and least by patients who used DA3. A full overview of the responses to perceived barriers and facilitators for all DA formats is presented in Table 3.

## Discussion and Conclusion

### Discussion

Many DA initiatives struggle to get structurally embedded in clinical routine, despite ample evidence revealing the benefits of using DAs when making medical decisions [7, 12]. At the onset of a multi-regional implementation initiative of three new Pca treatment DAs in Dutch clinical practice, a consortium was formed to jointly measure implementation rates and patient evaluations (i.e., barriers and facilitators from the patients’ perspective) from these three DAs. Overall, 40% of eligible Pca patients received a DA. For all DAs alike, implementation was quite successful (implementation rate > 80%) in a limited number of hospitals, whereas uptake varied widely at other sites (2–80%). Overall, patient evaluations were supportive of implementation of each DA; however, the online DA3 was evaluated as having the least facilitators.

The format of the implemented DAs as well as their level of information density varied [23–25]. DA1 and DA2 could be incorporated in clinical consultation, or used at home, while DA3 was, by design, supposed to be used outside of consultations. Despite the variation between DAs, implementation results showed the same variation between hospitals with each DA, and successful implementation (> 80%) was only achieved in a limited number of hospitals. Increasing the number of hospitals for implementation, as DA2 was implemented at 16 hospitals, compared with 8 and 9 hospitals for DA1 and DA3, did not result in more hospitals with successful implementation. This could suggest that for each DA, support was present in some hospitals prior to the start of implementation, and that for upscaling implementation, more structural encouragement and monitoring of implementation progress are needed in hospitals where the baseline support (in terms of care providers attitude or available resources) for DAs is lower.

When patient-perceived barriers were reported, most were related to DA characteristics (unpractical, unadjusted to needs) or expectations (no confidence, expected no benefits or reduction of uncertainty). Although overall report of barriers was low, barriers were reported most often for the online, elaborate DA3, and least for the very concise hybrid DA2. However, both DAs achieved similar implementation rates that were lower than the concise paper DA (DA1). This finding seems inconsistent with previous studies concluding that web-based DAs are the most promising modality for improving implementation [27, 28]. However, care providers have also shown hesitance towards online tools [29, 30]. Future research is needed to gain a deeper understanding of how the benefits of online tools, such as tailoring to patient information needs and enabling interactive VCEs, can be balanced with patients’ apparent preference for a more concise, paper format. One solution might be to provide concise, paper add-ons to online tools, which can be introduced during consultation and may enhance the user-friendliness of online tools.

The joint implementation efforts by the JIPPA consortium may have contributed to raising national awareness for SDM in both urology and oncology in The Netherlands. Many care providers have been introduced to the DA and to the principles of SDM, and during the course of the projects, consortium members contributed to national Pca treatment guidelines with a section on SDM and DAs (www.oncoline.nl). Therefore, the study in itself increased awareness for SDM and the existence of DAs and educated many teams in using DAs in clinical routine. However, it may also have caused a barrier, as clinical practice was unclear about which DA should be applied, and what the differences between the available DAs entailed. To the best of our knowledge, no earlier studies have reported (national) implementation rates for Pca DAs, and comparability to other DA implementations studies is difficult to interpret as they were aiming at different patient populations (e.g., women with breast cancer, or orthopedic patients) and settings (e.g., screening decisions often include the general practitioner) [10, 11, 31]. Further research is needed to determine if having different types of DA can help implementation since patients and care providers can select the DA they prefer most, or that the variety in available DAs hinders implementation since each DA has its specific characteristics and usability aspects that require training. Moreover, future research could study if specific DA characteristics have an effect on implementation rates, by randomizing distribution of different DA types across hospitals.

A strength of the current study was that we were able to investigate implementation of three DAs by using a similar questionnaire at a similar point in time. As a consequence of studying three different DAs, sample size and number of participating hospitals were higher than most previous Pca DA studies [9, 32]. Eventually, one in three Dutch hospitals was exposed to one of the three DAs. Hospitals from different levels (academic and non-academic) and from different regions were included in the study, increasing the generalizability of our findings.

A limitation of the current study is that the implementation rate was calculated based on actual receivers of a DA as proportion of an estimation of the total number of eligible patients. Since the number of patients eligible for study inclusion was not systematically registered by the hospitals included in our studies, we relied on the hospital-specific retrospective cohorts of PCa patients from the cancer registry. This ensured the sample was determined via the same method for every hospital. However, since the total number of patients eligible for DA receipt was estimated, this entailed that no information was available about patient characteristics from those patients who were possibly eligible but were not offered a DA. In particular, in hospitals with low implementation rates, a selection bias could have occurred if only patients were included who favored DA use. Another limitation is that the implementation period was not exactly simultaneous for all three DAs. Implementation of DA1 started almost a year ahead of DA2 and DA3. Moreover, a previous version of DA1 was studied in an earlier trial, which could have helped achieving the higher overall implementation of DA1 [25]. Furthermore, each participating hospital was linked to one of the three regions, and consequently implemented its respective DA. Possibly, some patients or care providers could have been more supportive of another DA and overall DA uptake would have been higher if all formats would be matched according to patient or care providers’ preferences. For example, one patient might benefit more from an elaborate DA, while for another patient, optimal understanding and satisfaction are reached with a concise DA [33–36]. Finally, no information was available from patients who received, and possibly also used, a DA but did not consent to participate in the survey study.

Patient evaluations from the three DAs in the current study were all favorable towards implementation. To further understand the observed differences in implementation rates between hospitals, future steps towards sustained DA use should include further investigation into barriers at the level of care providers and organizational barriers.

### Conclusion

Overall implementation rate of the DAs in clinical routine was 40%. A wide variation in uptake across hospitals was observed for each DA. Most patients were satisfied with the DA they received, and only few barriers of usage were perceived by patients. Offering an online-only DA led to less patient-reported facilitators compared with a paper-only or hybrid DA.

### Practice Implications

Patients appeared to be satisfied with each DA format. Sustained implementation of DAs in clinical routine requires further encouragement and attention, and could require a tailored distribution approach per hospital site [37].
